# Case Report: A rare case of synchronous ovarian mixed germ cell tumor and mast cell leukemia in a pediatric patient

**DOI:** 10.3389/fonc.2025.1717065

**Published:** 2026-01-06

**Authors:** Chengzhu Liu, Jinhua Chu, Yang Wan, Huiping Wang, Hongzhen Yu, Kunlong Zhang, Zhiwei Xie, Songji Tu, Ningling Wang, Linhai Yang

**Affiliations:** 1 Department of Hematology and Oncology in Pediatric, The Second Affiliated Hospital of Anhui Medical University, Hefei, China; 2 Department of the Hematologic Disease Diagnosis and Treatment Center, The Second Affiliated Hospital of Anhui Medical University, Hefei, China; 3 Department of Hematological Lab, The Second Affiliated Hospital of Anhui Medical University, Hefei, China; 4 Department of Pathology, The Second Affiliated Hospital of Anhui Medical University, Hefei, China

**Keywords:** germ cell tumor, KIT D816V, mast cell leukemia, pediatric, systemic mastocytosis

## Abstract

Patients with concurrent malignancies pose significant diagnostic and therapeutic challenges. We report a rare and fatal case of synchronous ovarian mixed germ cell tumor and mast cell leukemia (MCL) in a 13-year-old female, characterized by a shared clonal origin. The patient initially presented with a large pelvic mass, elevated alpha-fetoprotein(AFP) and human chorionic gonadotropin (β-HCG), anemia, and thrombocytopenia. Exploratory laparotomy confirmed a diagnosis of mixed germ cell tumor, predominantly dysgerminoma with a minor choriocarcinoma component. Despite an initial decrease in serum tumor markers to platinum-based chemotherapy, persistent cytopenias and bone marrow infiltration raised concern for hematologic malignancy. Genomic analyses of both ovarian tumor and bone marrow samples identified identical somatic mutations, including KIT D816V, NRAS G12C and TP53 Y220C, strongly suggesting a common progenitor. Subsequent immunophenotyping, histology, and transcriptome sequencing confirmed the diagnosis of concurrent mast cell leukemia. Targeted therapy with avapritinib and ruxolitinib was initiated but yielded limited response. Further salvage therapy failed due to disease progression and treatment intolerance, and the patient succumbed to multiple organ failure. This case underscores the clinical and genetic overlap between germ cell tumors and hematological malignancies in pediatric patients, highlighting the role of KIT mutations as a potential unifying driver. Given the consistent co-occurrence of KIT mutations in previously reported similar cases, we propose the recognition of a distinct disease entity: ovarian germ cell tumor/mastocytosis with KIT mutations. This report emphasizes the importance of early genetic profiling and multidisciplinary collaboration in diagnosing and managing rare, genetically unified malignancies in pediatric oncology.

## Introduction

It is exceedingly rare for pediatric patients with solid tumors to also present with concurrent hematological malignancies. Even less is understood about synchronous malignancies arising from a common clonal origin (1). To date, there have been few reports of germ cell tumors (GCTs) occurring simultaneously or subsequently to systemic mastocytosis (SM). These cases are typically characterized by female sex, pediatric-onset, neoplastic proliferation in both gonad and bone marrow and all accompanied by *KIT* mutations (1–4). It constitutes a highly homogeneous clinical and genetic disease entity. Although mutations in *KIT* are recognized as key drivers of clonal evolution in both germ cell tumor and hematological malignancies, the clinical and genomic interactions between GCT and SM remain largely unexplored, particularly in the pediatric population (4). Unfortunately, research into human embryonic hematopoiesis faces significant ethical challenges. Therefore, how to identify such patients early and give accurate diagnosis is particularly important.

Here, we report a novel case of synchronous ovarian mixed germ cell tumor and mast cell leukemia (MCL) in a pediatric patient. Targeted DNA next-generation sequencing analysis revealed that the same genomic changes were present in both tissues, including the same *KIT D816V* mutation. This indicates that they share a common progenitor cancer cell. It highlights the challenges in the nosological classification of this type of tumor. It reflects the still poorly understood pathophysiology of mixed tumors.

## Case description

**Figure 1:**
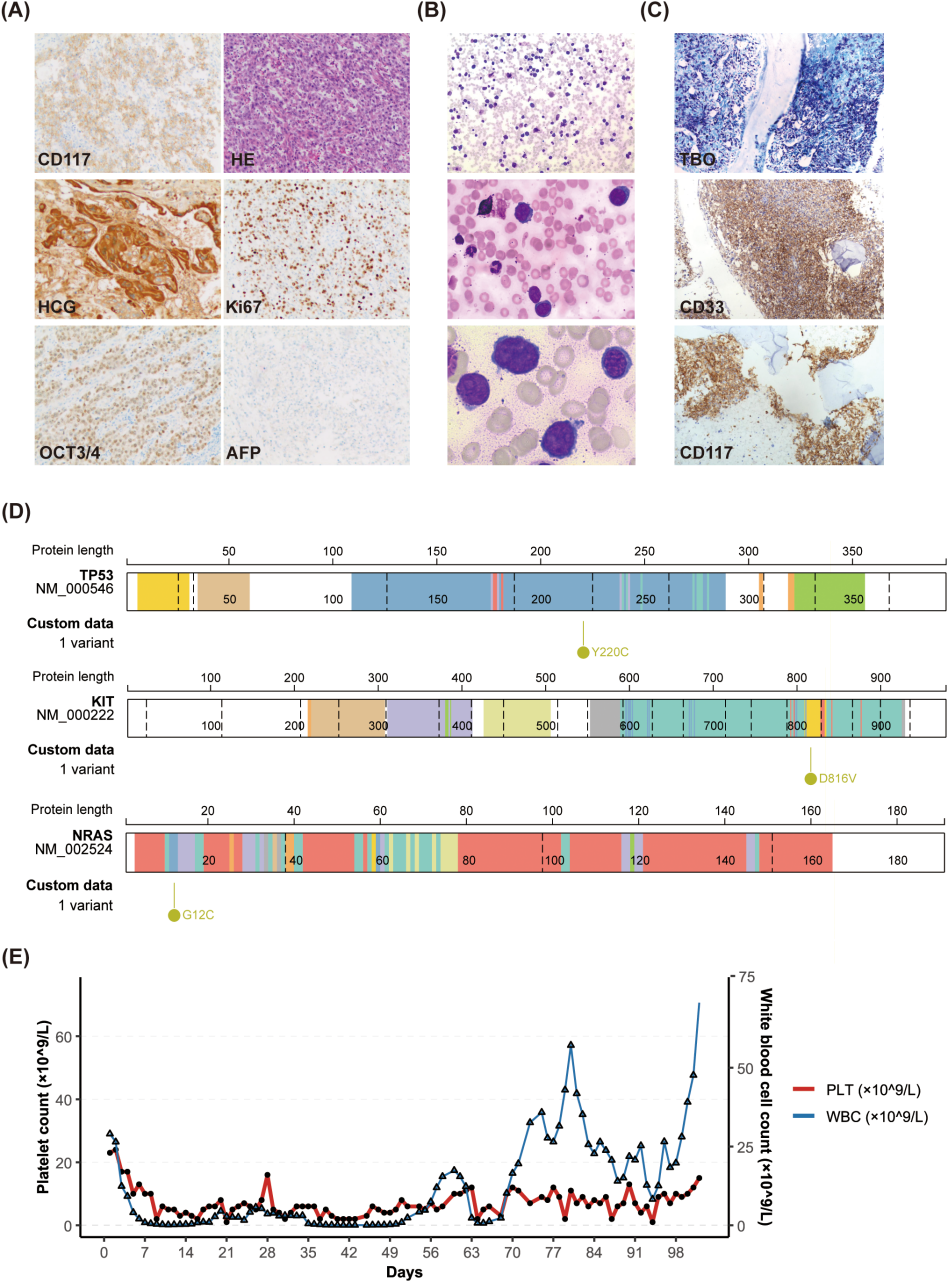
The main laboratory test results and clinical course of this patient. **(A)** The ovarian tumor tissues immunohistochemical results of the child showed that OCT3/4, HCG, CD117 and Ki67 were positive, and AFP was negative. **(B)** Wright-giemsa staining showed abnormal cells in bone marrow smears. From top to bottom, they are 10×,20× and 100×. **(C)** Some of the immunohistochemical results of the patient’s bone marrow tissue showed positive staining for TBO, CD33 and CD117. **(D)** Diagram of the genetic mutation site of the patient. Conserved protein domains (predicted by Pfam, https://pfa-m.xfam.org/) are color coded and shown in the accompanying key. Protein diagrams were generated using Protein Paint (https://pecan.stjude.cloud/variants/proteinpaint) (17). For more detailed illustrations, please refer to **Supplementary Figure S4**. **(E)** Changes of white blood cells and platelets in peripheral blood of this child over time.

The patient was a 13-year-old female with no notable personal or family history of malignancy. She initially presented at an outside hospital because of nausea, fatigue, and fever. Computed tomography (CT) of the abdomen and pelvis revealed a large mass occupying the abdominal and pelvic cavities. During empiric anti-infective therapy with cefazolin, the patient developed an anaphylactic shock despite no prior history of hypersensitivity to cephalosporins. She was subsequently transferred to our institution on May 28, 2024. At admission, CT imaging demonstrated a pelvic mass measuring approximately 150.2×106 mm, alongside splenomegaly. Cranial magnetic resonance imaging (MRI) including diffusion-weighted imaging (DWI) and whole-body bone scintigraphy yielded unremarkable findings. Laboratory tests showed anemia (hemoglobin 91 g/L) and thrombocytopenia (platelet count 74 × 10^9^/L). Tumor markers were markedly increased, including alpha-fetoprotein (AFP) at 801 ng/mL(ref. 0-7.00 ng/mL), and human chorionic gonadotropin (β-HCG) at 15,491 mIU/mL(ref. 0-5.00 mIU/mL). These findings raised a strong clinical suspicion for a germ cell tumor. On May 30, 2024, the patient underwent exploratory laparotomy, which revealed an irregular, cystically enlarged left ovary measuring approximately 20×12×10cm. Immunohistochemical staining showed positivity for OCT3/4, SALL4, CD117, D2–40 and HCG, whereas markers including AFP, glypican-3 (GPC3) and CDX-2 were negative (**Figure 1A**, **Supplementary Figure S1**). Based on these findings, a diagnosis of ovarian mixed germ cell tumor was established, predominantly comprising dysgerminoma with a minor non-gestational choriocarcinoma component. Whole genome sequencing (WGS) of the tumor was performed on the same day. Given the patient’s anemia and thrombocytopenia, there was a strong clinical suspicion of bone marrow metastasis. Therefore, bone marrow examination was performed from bilateral posterior superior iliac spines. Bone marrow cytology of both sides showed abnormal cells, about 51% on the left side and 49% on the right side (**Figure 1B**). Histopathological analysis confirmed extensive infiltration of tumor cells within the bone marrow (**Supplementary Figure S2**).

On June 6, 2024, chemotherapy was initiated following the PEB regimen (etoposide (VP-16) 100 mg/m² day 1-5, cisplatin (CDDP) 20 mg/m² day 1-5, and bleomycin (BLM) 15 mg/m² day 1) outlined in the CCCG-GCTs-2021 protocol. Serial monitoring demonstrated a progressive decline in serum AFP and β-HCG levels (**Supplementary Figure S3**). By June 20, serum β-HCG declined to below the assay’s detection limit (<2.39 mIU/mL). Nevertheless, despite hematologic recovery from myelosuppression, the patient continued to exhibit severe anemia and profound thrombocytopenia (**Figure 1E**). On July 1, 2024, whole genome sequencing of the primary tumor tissue uncovered three clinically relevant somatic mutations: *KIT* (exon17:C.2447A>T:p.D816V, variant allele frequency(VAF): 42.74%), *NRAS* (exon2:c.34G>T:p.G12C, VAF: 2.85%) and *TP53* (exon6:c.659A>G:p.Y220C, VAF:2.43%) (**Figure 1D**, **Supplementary Figure S4**, **Supplementary Table S1**). In accordance with current guidelines, targeted therapy with avapritinib (100 mg once daily) and ruxolitinib (5 mg twice daily) was subsequently incorporated into the treatment regimen (6–9). The child underwent chemotherapy with the C-PEB regimen (CTX 1.2 g/m² day 1, VP-16–100 mg/m² days 1-5, CDDP 20 mg/m² days 1-5, BLM 15 mg/m² day 1) on July 8, 2024, in conjunction with targeted oral therapy. Throughout this treatment, HCG levels remained within the normal range (**Supplementary Figure S3**). However, daily physical examinations indicated persistent splenomegaly, and ongoing transfusion support was required for her platelet and red blood cell counts. A repeat bone marrow aspiration was performed on July 30, 2024, and there was still a large amount of tumor cell infiltration. Immunohistochemical and histochemical analyses indicated diffuse proliferation of spindle cells (mast cells). Flow cytometric immunophenotypic studies demonstrated that the blasts were positive for CD34 (99.8%),CD13 (12.1%),HLA-DR (36.4%), CD117(55.7%),CD33(99.8%) but negative for MPO, cCD79a, cCD22, cCD3 (**Supplementary Table S2**). The mutation frequency of KIT D816V in bone marrow, as assessed by Digital-PCR, remained at 46.42%. This is in contradiction to the completely normal AFP and HCG levels of this patient. Given the lack of response to PEB and CPEB chemotherapy, and the high likelihood of resistance to avapritinib, we suspected the presence of a concurrent hematologic malignancy instead of simple bone marrow metastasis from the germ cell tumor. Consequently, we performed bone marrow whole transcriptome sequencing to confirm the diagnosis and investigate potential effective chemotherapy regimens. On August 14, 2024, bone marrow whole transcriptome sequencing confirmed the persistent co-occurrence of *KIT D816V*, *NRAS G12C*, and *TP53 Y220C* mutations at identical variant loci (**Figure 1D**, **Supplementary Table S3**). Due to the rare nature of this case, in order to further clarify the diagnosis, we reviewed the previous bone marrow tissues under a joint multidisciplinary consultation. We found that the bone marrow was characterized by diffuse infiltration of abnormal mast cells, accounting for about 80% of the cells in the bone marrow, with more than 15 mast cells per high-power field. The serum tryptase level was measured at 34.6 ng/mL, which is above the normal reference range. Immunohistochemical analysis revealed positivity for CD25, CD117, and CD33 as well as toluidine blue (TBO) staining (**Figure 1C**, **Supplementary Figure S2**). Finally, we diagnosed mixed ovarian germ cell tumor (stage IV) with mast cell leukemia.

To identify potentially effective chemotherapy options, high-throughput drug sensitivity assays were conducted (**Supplementary Table S4**). After comprehensive test results and obtaining written informed consent from the patient’s family, treatment with the VTD regimen (bortezomib 1.3 mg/m², thalidomide 200 mg, dexamethasone 20 mg, and arsenic trioxide (As_2_O_3_) 0.15 mg/kg) was started on September 2, 2024. Five days later, the patient developed progressively worsening sensory and perceptual disturbances in her limbs, raising suspicion for central nervous system leukemia. However, due to severe thrombocytopenia, lumbar puncture was contraindicated. Consequently, chemotherapy was discontinued on September 8, 2024, and only palliative management with oral avapritinib and hydroxyurea was continued. During this period, peripheral white blood cell counts increased rapidly (**Figure 1E**). Unfortunately, the patient succumbed to multiple organ failure on September 19, 2024.

## Discussion

This is a rather rare pediatric case of synchronous ovarian germinoma and mast cell leukemia, linked by a common somatic mutation of *KIT D816V*, along with co-mutations of *NRAS G12C* and *TP53 Y220C*. These two histologically unrelated tumors harbor the same malignant clone, consistent with a common primitive cancer cell origin. *KIT* mutation is a marker of mastocytosis and exists in more than 90% of systemic mastocytosis cases (6, 10). *KIT* mutations are also found in approximately one-third of teratomas or seminomas, indicating that *KIT* signaling plays a key role in the development of germ cell tumors (11, 12). The co-occurrence of *NRAS* and *TP53* mutations may further exacerbate genomic instability and resistance to conventional therapies. Recent studies suggest that these mutations can promote immune evasion, stemness, and treatment resistance across tumor types (5, 14, 15). In the latest guidelines for the treatment of SM/MCL, the first-line recommended targeted drug is avapritinib (6–9, 16). Despite the introduction of avapritinib targeted therapy, the desired therapeutic outcome was not achieved.

Anaphylactic shock is a life-threatening systemic allergic reaction that can be triggered by various agents, including foods, medications, and insect stings. In patients with mast cell disorders, pathological expansion of mast cells can lead to heightened responsiveness to allergens, thereby increasing susceptibility to severe anaphylactic episodes. Notably, this patient had no prior history of cephalosporin allergy but developed severe anaphylactic shock following administration of a cephalosporin at an outside hospital. Given the subsequent diagnosis of systemic mastocytosis and detection of the KIT D816V mutation, we infer that clinically occult mast cell proliferation was already present at the time of the reaction. This case underscores the importance of maintaining a high index of suspicion for underlying mast cell disease in pediatric patients presenting with unexplained anaphylaxis, even in the absence of known risk factors.

Several previous studies have described similar cases, all of which involved female patients with pediatric-onset, and the types of gonadal tumors included mixed germ cell tumors, teratomas, and atypical gonadal tumors. All patients presented with mastocytosis. The majority of these patients exhibited KIT mutations, including the hotspot D816 mutation. Given the high similarity in clinical presentation and the co-occurrence of KIT mutations, we also propose that these diseases be classified as a genetically highly homologous disease entity, namely ovarian germ cell tumors/mastocytosis with KIT mutations. We advocate for the inclusion of this classification and management strategy in future guidelines. Notably, the association between germ cell tumors and hematologic malignancies suggests that primitive germ cells may be the progenitors of hematopoietic stem cells (13). The genetic mutation characteristics observed in this case provide strong support for this hypothesis.

In conclusion, this rather rare case highlights the role of the *KIT D816V* mutation in combination with *NRAS* and *TP53* mutations in driving the malignancies of ovarian GCT and MCL, as well as the challenges it poses for diagnosis and treatment. This case adds to the limited knowledge base regarding the concurrent occurrence of germ cell tumors and hematological malignancies in pediatric patients and suggests that they may be part of a genetically homologous disease entity, requiring specialized management strategies. Future efforts should aim to explore combination therapies targeting shared driver mutations in both GCT and hematologic malignancies.

## Data Availability

The original data presented in the article are included in the article/supplementary materials. Further clinical data can be obtained by contacting the corresponding author directly.
