# Supplementary material for: Case Report: A rare case of synchronous ovarian mixed germ cell tumor and mast cell leukemia in a pediatric patient
Source: Front Oncol. 2026 Jan 6;15:1717065. doi: 10.3389/fonc.2025.1717065 (PMC12815785; doi:10.3389/fonc.2025.1717065)
Supplement: Supplementary file 1 [file DataSheet1.pdf]

Supplementary Material

Supplementary Figure1 Immunohistochemistry of ovarian tumor tissues……………………..2

Supplementary Figure2 Immunohistochemical study of bone marrow tissues………………..3

Supplementary Figure3 The trends of β-HCG and AFP, respectively…………………………4

Supplementary Figure4 Diagram of the genetic mutation site of the patient…………………5

Table.S1 Genetic testing of ovarian tumors (peCanDX Panel)…………………………………6

Table.S2 Bone marrow immunophenotyping results of the patient……………………………7

Table.S3 Whole transcriptome sequencing of bone marrow……………………………………8

Table.S4 Results of High-throughput Drug Sensitivity Analysis………………………………9

References ………………………………………………………………………………………11

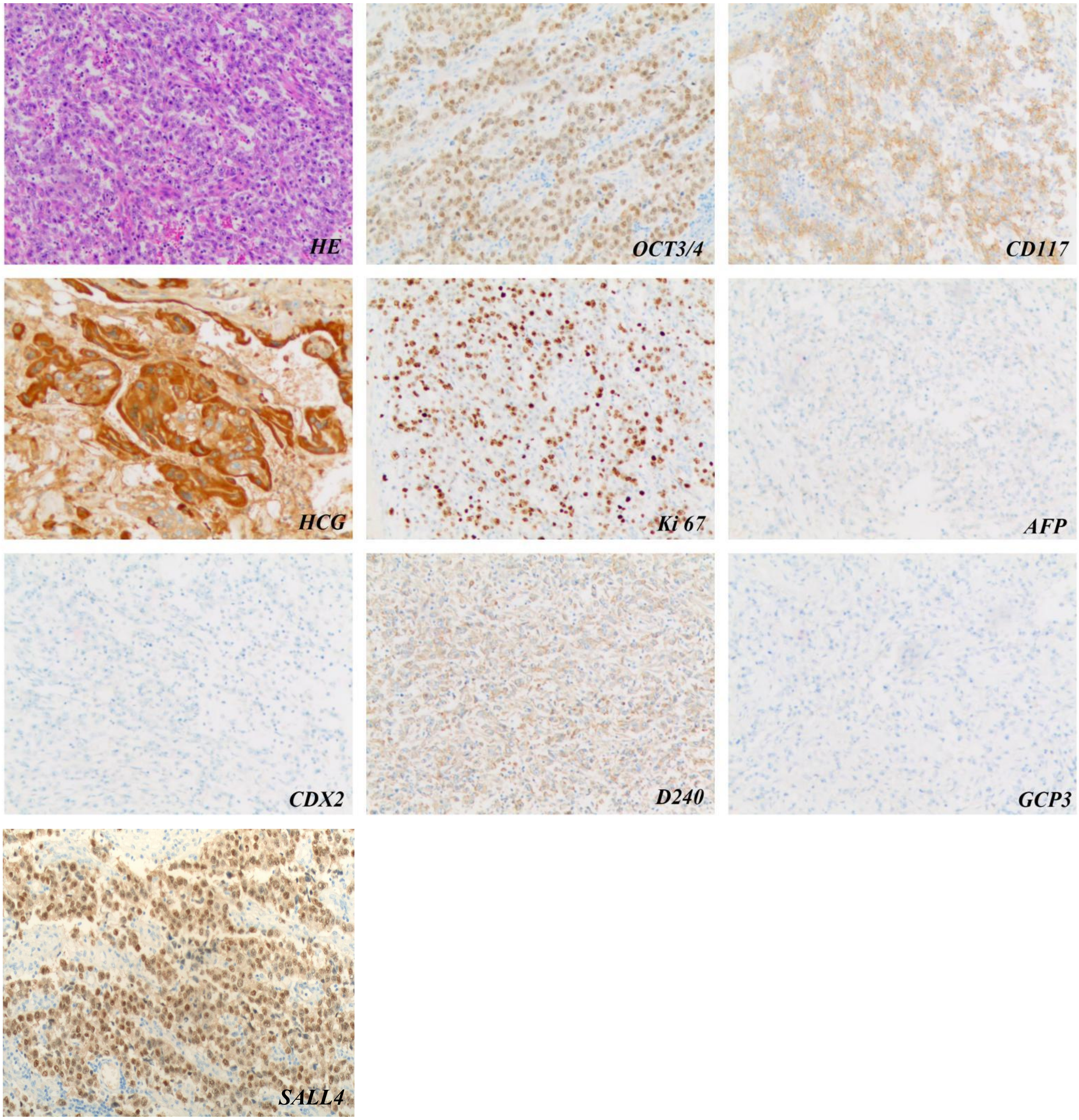

Supplementary Figure 1. Immunohistochemistry of ovarian tumor tissues.The magnification was 100X.

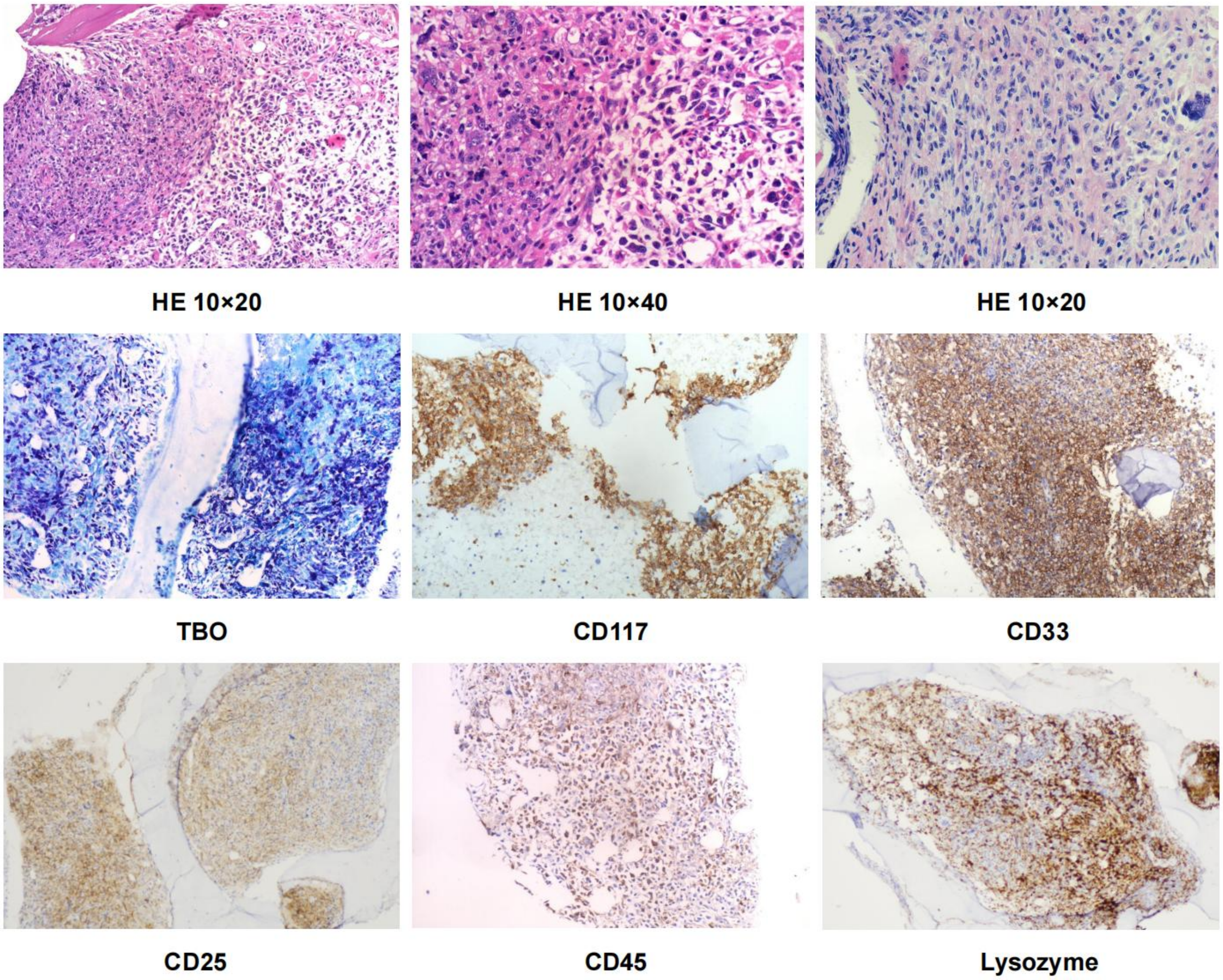

Supplementary Figure2 Immunohistochemical study of bone marrow tissues.

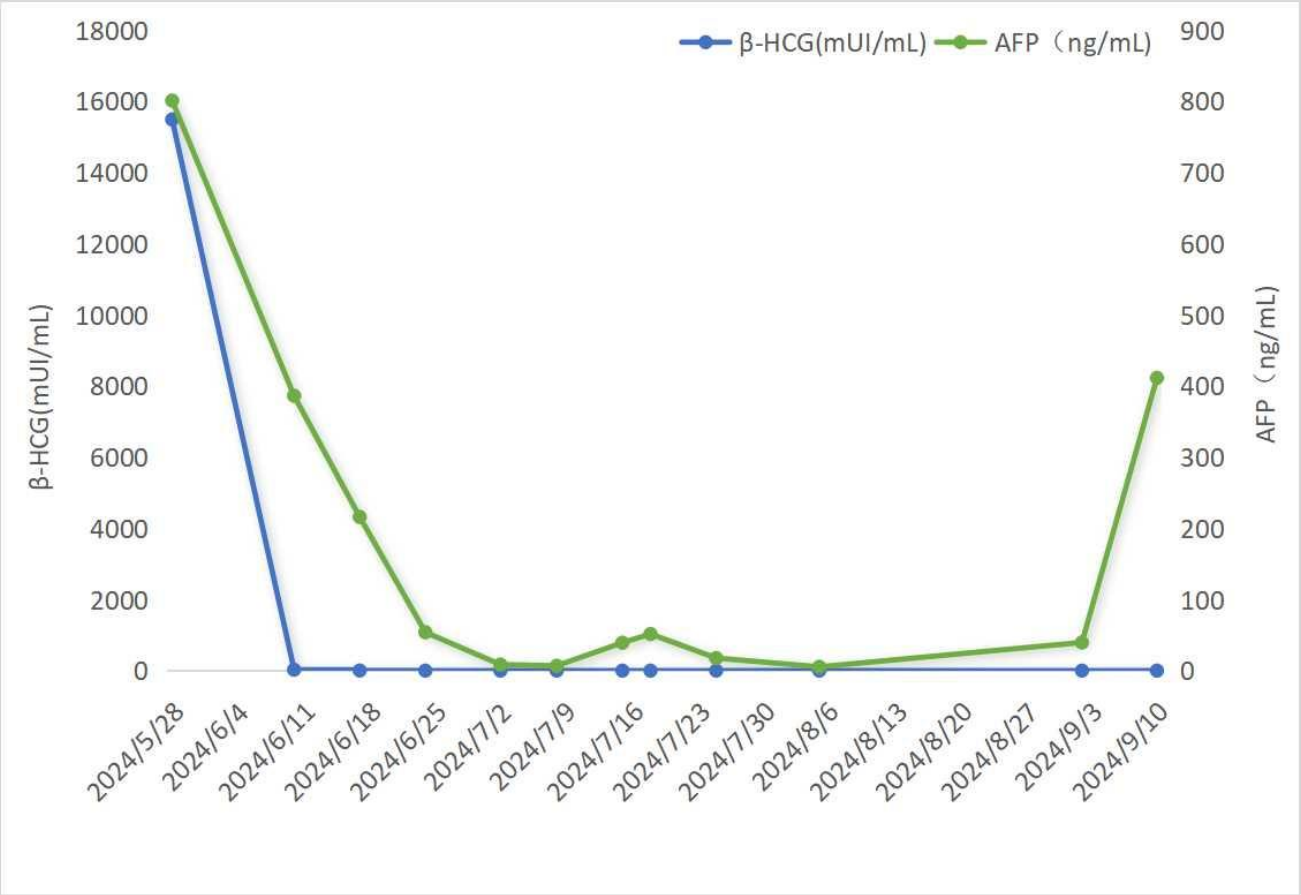

Supplementary Figure3 The trends of β-HCG and AFP, respectively

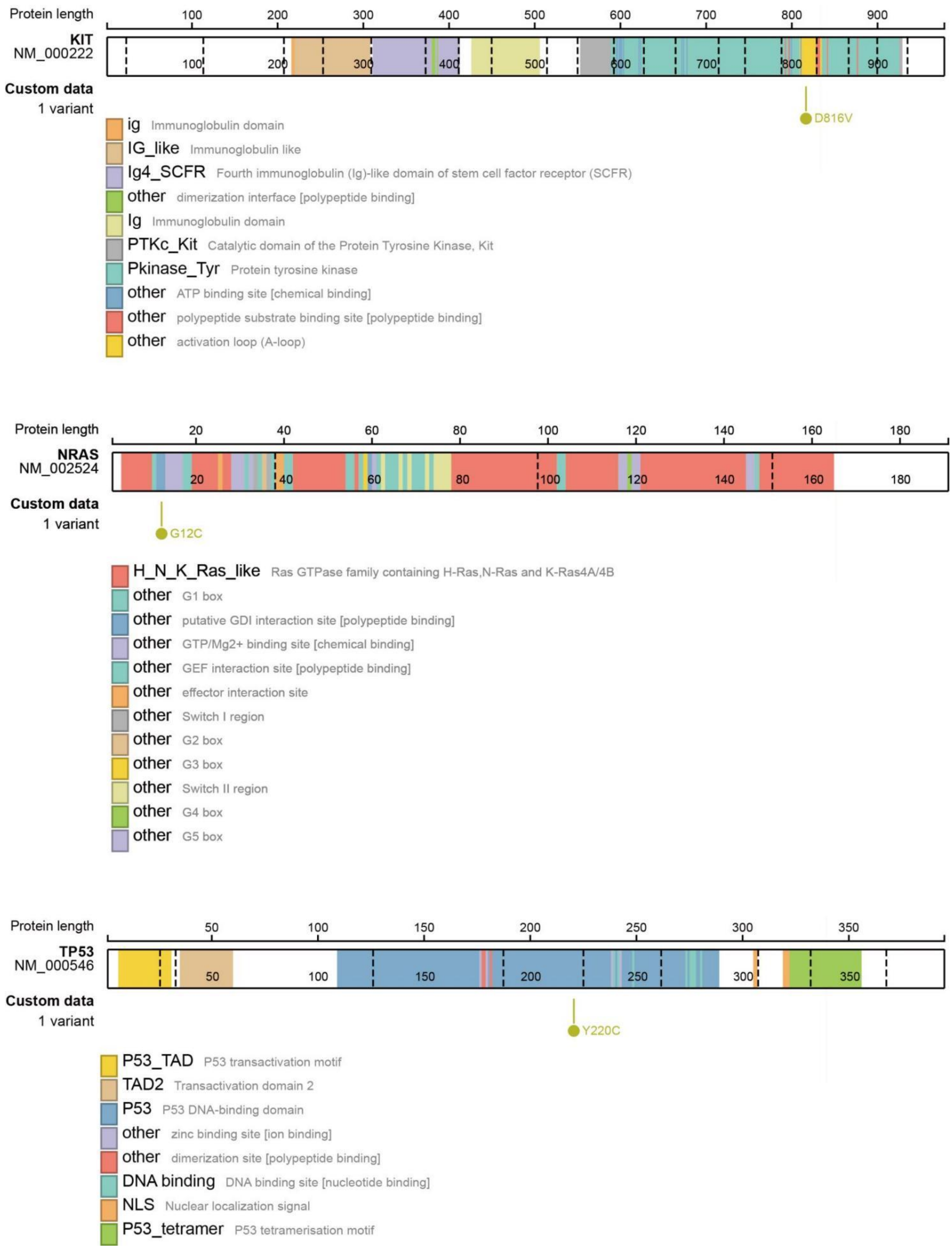

Supplementary Figure4 Diagram of the genetic mutation site of the patient

Table.S1 Genetic testing of ovarian tumors (peCanDX Panel)

Name	Location of chromosomes	Locus of variation	VAFs	Sources	Grade*
NRAS<br>NM_ 002524.5	chr1:115258748C>A	exon2:c.34G>T:p.G12C	2.85%	Somatic mutations	Level 1
KIT<br>NM_ 000222.3	chr4:55599321A>T	exon17:C.2447A>T:p.D816V	42.74%	Somatic mutations	Level 1
TP53<br>NM_ 000546.6	chr17:7578190T>C	exon6:c.659A>G:p.Y220C	2.43%	Somatic mutations	Level 1

Notes: VAFs: variant allele frequencies.

*Report interpretation: Tumor somatic mutations were interpreted according to the standards and guidelines for the interpretation of tumor genetic variants jointly issued by the American Society of Molecular Pathology (AMP), American Society of Clinical Oncology (ASCO) and Society of American Pathologists (CAP). The pathogenicity of germline mutation sites was graded according to the standards and guidelines for the interpretation of genetic variants published by the American College of Medical Genetics and Genomics (ACMG) and the Joint Society for Molecular Pathology (AMP) and the College of American Pathologists (CAP) in 2015.

Table S2. Bone marrow immunophenotyping results of the patient

B cell markers		T cell markers		Myeloid marker		Stem and progenitor cell markers and others	
CD	%	CD	%	CD	%	CD	%
CD19(APC)	0	CD7	45.0	MPO	0	CD34	99.8
cCD79a	0	cCD3	0	CD33	99.8	HLA-DR	36.4
cCD22	0	TCR a β	0	CD13	12.1	CD9	0
CD10	0	TCR γδ	0	CD11b	80.2	CD123	97.3
CD20	0	CD3	0	CD64	0	CD66c	0
CD22	0	CD4	53.5	CD36	0	CD56	15.7
smIgM	0	CD8	0	CD14	0	CD117	55.7
cu	0	CD1a	0	CD15	0	CD38	9.7
TDT	0	CD2	0	CD71	0	CD133	65.8
CD79b	0	CD5	0	CD61	0	NG2	0
				GlycophorinA:	0		
				CD41	0		
				CD65	0		

Table.S3 Whole transcriptome sequencing of bone marrow

Name	Information of variation	Location of chromosomes	VAFs	Grade*
NRAS	NM_ 002524.5: exon2:c.34G>T:p.G12C	chr1:115258748C>A	98.00%	Level 1
KIT	NM_ 000222.3: exon17:C.2447A>T:p.D816V	chr4:55599321 A>T	65.10%	Level 1
TP53	NM_ 000546.6: exon6: c.659A>G:p.Y220C	chr17:7578190 T>C	93.80%	Level 1

*According to the standards and guidelines for the interpretation of tumor gene variants issued by the Society of Molecular Pathology of America (AMP), American Society of Clinical Oncology (ASCO) and Society of American Pathologists (CAP), the mutation grades are divided into the following 3 categories :

Level1 refers to mutations with clear clinical significance, including :levelA, drug treatment or drug resistance targets approved by the State Food and Drug Administration (NMPA), the US Food and Drug Administration (FDA) and other agencies; levelB, based on expert consensus or authoritative literature repeated reports of mutations with diagnostic, therapeutic, and prognostic significance in pediatric solid tumors.

Leve2: mutations with potential clinical significance, including :levelC, mutations that have been reported to have diagnostic, therapeutic or prognostic significance in pediatric solid tumors based on several small studies, but no consensus has been reached; levelD, a variant associated with a therapeutic target in preclinical trials or a newly identified somatic mutation in an important domain of a disease-related gene.

Leve3: mutations of uncertain clinical significance, including low distribution frequency in the population and no reported SNP of the patient itself.

Table.S4 Results of High-throughput Drug Sensitivity Analysis

H D S test results collection					
		**Highly sensitive**	**Medium sensitivity**	**Low sensitivity**	**Insensitive**
**Cytotoxic drugs**	**Alkylating Agents**		Ifosfamide ($2500mg/m^2$)		Dacarbazine,Bendamustine,Chlorambucil,Busulfan, Cyclophosphamide,Lomustine,Ifosfamide($1200mg/m^2$),Melphalan,Carmustine,Procarbazine
	**Antimetabolites**	—	Gemcitabine	Cytarabine($3000mg/m^2$、$2000mg/m^2$), Hydroxyurea (60mg/kg), Fludarabine	Cytarabine($100mg/m^2$), Azacitidine,Hydroxyurea(20mg/kg),Methotrexate,Clofarabine,Decitabine,Cladribine,6-Mercaptopurine,5-Fluorouracil
	**Microtubule Inhibitors**	—	—	Vinblastine,Vindesine,Paclitaxel,Docetaxel	Vinorelbine, Vincristine
	**Topoisomerase Inhibitors**			Hydroxycamptothecin,Irinotecan	Teniposide, Topotecan,Etoposide (VP-16)
	**Antibiotics**		Mitoxantrone	Epirubicin($120mg/m^2$),Daunorubicin, MitomycinC(20mg)	Aclarubicin,Epirubicin ($60mg/m^2$),Doxorubicin,Pirarubicin,Idarubicin,**Bleomycin**,MitomycinC(10mg)
	**Antiviral agent**	—	—	—	Adefovir Dipivoxil
	**Others**		As_2O_3	Homoharringtonine (4mg),Bexarotene, Deferasirox,Deferoxamine,All-trans Retinoic Acid (ATRA,Tretinoin)	Homoharringtonine(1mg), **Cisplatin**, Amsacrine,Oxaliplatin, **Carboplatin**
**Adjunctive Therapies**	**Glucocorticoids**	—	—	—	Methylprednisolone,Hydrocortisone,Prednisone,Dexamethasone
	**Immunomodulator**	—	—	Thalidomide, lenalidomide	Pomalidomide, Mycophenolate Mofetil

**Targeted drugs**		Bortezomib, carfilzomib	Ponatinib	**Avapritinib,** Dasatinib Chidamide,**Ruxolitinib** Venetoclax,MidostaurinNilotinib,Bosutinib, Imatinib,Ixazomib, Ibrutinib
**Chemotherapy regimens**	VTD<br>MA<br>ICE	HDMTX+L-ASP,VDLP,VDLD,VDCLP,Selinexor+Dex,MOAP,MOACD,MINE,ME,IOLP,Hyper-CVAD(B),GDP,FLAG,DOLP,DAT,CLAG	HOAP,HD-DA,HAD,HAA,HA,ESHAP,DOMP,DHAP,DAE,DAC,DA,COATD,CAM	TA,Mm,IOAP,IAE,IA,Hyper-CVAD(A),DCAG,DA-EPOCH,COP,CHOP,CHOEP,CAG,ABVD,6-MP+MTX
**Highly sensitive chemotherapy regimens**				
**ICE**	VP-16 100 mg/m²<br>Carboplatin 800 mg<br>Ifosfamide 5 g/m²		Rate of inhibition 93.23%	Highly sensitive
**VTD**	Bortezomib 1.3 mg/m²<br>Thalidomide 200 mg/d<br>Dexamethasone 20 mg		Rate of inhibition 86.90%	Highly sensitive
**MA**	Mitoxantrone 12 mg/m²<br>Cytarabine 200 mg/m²		Rate of inhibition 81.94%	Highly sensitive

Notes:

Sensitivity grades (high, medium, and low) represent only the results of drug evaluation at corresponding clinical doses.

Inhibition ratio: evaluation of the killing effect of the drug on the subject's autologous cancer cells at 100% PPC screening concentration, only represents the drug at the corresponding clinical dose. The higher the inhibition rate, the stronger the effect, indicating that the patient's autologous tumor cells were more sensitive to the drug.

Inhibition Rate (%) = (1 - Number of viable tumor cells in the drug-treated group / Number of viable tumor cells in the blank control group) × 100%

Highly sensitive: Rate of inhibition ≥ 80%

Medium sensitivity: 50% ≤ Rate of inhibition < 80%

Low sensitivity: 20% ≤ Rate of inhibition < 50%

Insensitive: Rate of inhibition < 20%

References

1. Richards S,Aziz N,Bale S,Bick D,Das S,Gastier-Foster J,Grody WW,Hegde M,Lyon E,Spector E,Voelkerding K,Rehm HL, .Standards and guidelines for the interpretation of sequence variants: a joint consensus recommendation of the American College of Medical Genetics and Genomics and the Association for Molecular Pathology.Genet Med.2015 May;17(5):405-24.doi:10.1038/gim.2015.30.PMID25741868.

2. Li MM,Datto M,Duncavage EJ,Kulkarni S,Lindeman NI,Roy S,Tsimberidou AM,Vnencak-Jones CL,Wolff DJ,Younes A,Nikiforova MN.Standards and Guidelines for the Interpretation and Reporting of Sequence Variants in Cancer: A Joint Consensus Recommendation of the Association for Molecular Pathology, American Society of Clinical Oncology, and College of American Pathologists.J Mol Diagn.2017 Jan;19(1):4-23.doi:10.1016/j.jmoldx.2016.10.002.PMID27993330.
